# Monitoring the ability to deliver care in low- and middle-income countries: a systematic review of health facility assessment tools

**DOI:** 10.1093/heapol/czu043

**Published:** 2014-06-03

**Authors:** Jason W Nickerson, Orvill Adams, Amir Attaran, Janet Hatcher-Roberts, Peter Tugwell

**Affiliations:** ^1^Institute of Population Health, University of Ottawa, Ottawa, ON, Canada, ^2^Bruyère Research Institute, Ottawa, ON, Canada, ^3^Orvill Adams and Associates, Ottawa, ON, Canada, ^4^Faculty of Common Law, University of Ottawa, Ottawa, ON, Canada, ^5^Faculty of Medicine, University of Ottawa, Ottawa, ON, Canada, ^6^Canadian Society for International Health, Ottawa, ON, Canada and ^7^Ottawa Hospital Research Institute, The Ottawa Hospital, Ottawa, ON, Canada

**Keywords:** Health system, health facilities, methodologies, health services accessibility, health care surveys

## Abstract

**Introduction** Health facilities assessments are an essential instrument for health system strengthening in low- and middle-income countries. These assessments are used to conduct health facility censuses to assess the capacity of the health system to deliver health care and to identify gaps in the coverage of health services. Despite the valuable role of these assessments, there are currently no minimum standards or frameworks for these tools.

**Methods** We used a structured keyword search of the MEDLINE, EMBASE and HealthStar databases and searched the websites of the World Health Organization, the World Bank and the International Health Facilities Assessment Network to locate all available health facilities assessment tools intended for use in low- and middle-income countries. We parsed the various assessment tools to identify similarities between them, which we catalogued into a framework comprising 41 assessment domains.

**Results** We identified 10 health facility assessment tools meeting our inclusion criteria, all of which were included in our analysis. We found substantial variation in the comprehensiveness of the included tools, with the assessments containing indicators in 13 to 33 (median: 25.5) of the 41 assessment domains included in our framework. None of the tools collected data on all 41 of the assessment domains we identified.

**Conclusions** Not only do a large number of health facility assessment tools exist, but the data they collect and methods they employ are very different. This certainly limits the comparability of the data between different countries’ health systems and probably creates blind spots that impede efforts to strengthen those systems. Agreement is needed on the essential elements of health facility assessments to guide the development of specific indicators and for refining existing instruments.

**KEY MESSAGES**

- A large number of health facility assessment tools currently exist, using different methodologies and indicators and limiting the comparability and comprehensiveness of health systems data.
- Consensus is needed on specific indicators for monitoring the capacity and functionality of health facilities in low- and middle-income countries.

## Introduction

The need to move beyond individual health services and strengthen health systems has become a critical component of global public health and international development (Frenk 2010; Samb *et al.* 2010; Atun 2012). Compared to a decade ago, there is renewed emphasis on horizontal health systems strengthening rather than vertical, disease-oriented programming in low- and middle-income countries (LMICs) (Task Force on Health Systems Research 2004; Travis *et al.* 2004; Adam and de Savigny 2012; Swanson *et al.* 2012).

Most would agree that to improve population health, health services must be available, accessible, efficacious and used by the population. To achieve this, comprehensive interventions are needed that strengthen not only service delivery, but also the laws and policies that influence the functionality of a health system and the health-seeking behaviour of the population. To achieve those reforms, one needs a pragmatic assessment of where gaps or weaknesses exist within the system using rigorous and valid methodologies to determine the above-listed factors. Collecting data at the level of health facilities allows for a detailed assessment of the various components that function (or do not) at the level of service delivery, which is a useful level of analysis for identifying the weaknesses within a national health system (Paxton *et al.* 2006).

The outputs of these assessments provide important data to guide further health systems planning, such as the resources available within geographic areas and the proximity of essential health services to higher levels of care (Gabrysch *et al.* 2011). Thus, research for development and evidence-based planning and policy making are integral for understanding the distribution of health services and also for estimating and ensuring the effective coverage of various health services (Shengelia *et al.* 2005).

Yet how one should conduct assessments of health systems at the health facility level is currently controversial and disorganized. Broad frameworks such as World Health Organization’s (WHO) health systems building blocks (World Health Organization 2007) or health systems research and evaluation (Murray and Frenk 2000; International Health Partnership + Related Initiatives and World Health Organization 2011) have been developed in part to provide guidance and some standardization in language and concepts, while retaining the flexibility needed to adapt to different countries and contexts. Yet evidence suggests that when evaluations of health systems interventions are conducted, they often do not adhere to these basic frameworks or standards, which are arguably more aspirational than real (Adam *et al.* 2012). Not surprisingly, without such standardization, there is also a controversy as to whether new evidence and research findings obtained in one country can be applied in another remain. (Task Force on Health Systems Research 2004). There are also lacunae in the health system assessments that are done, ranging from incomplete indicators in assessments to the outright lack of pivotal data such as a master list of health facilities (Falade *et al.* 2006; Casey *et al.* 2009).

The fact that health facility assessments are often patchy or incomplete is problematic for achieving important health gains in LMIC. Major investments in initiatives to scale up health services necessarily rely on reliable, accurate and comprehensive information on both capacities and gaps in health services availability to prioritize interventions and ensure equitable access to care. To achieve this, there is a need to ensure the development of evidence-based policies and interventions to improve the performance of health systems in LMIC (Bosch-Capblanch *et al.* 2012). Poor quality assessments are likely to impede this and lead to resource misallocation and therefore inferior health outcomes. Not only is this anathema to the central tenet that a country’s health system should be structured in evidence-based ways, but it likely impedes progress on global initiatives such as the Millennium Development Goals (Attaran 2005) when necessary measurements are not made, sometimes in a majority of countries.

This study asked whether the vagaries of health system assessments have a common root: the quality and comprehensiveness of health facility assessment tools. To answer this question, we performed a comparative analysis of the different tools that are currently used to assess the administrative and service delivery capabilities of health facilities in LMICs, charting their similarities and differences. We hypothesized that if a genuine consensus on health system strengthening existed, then the assessment tools used to assess the capabilities at the point of actual service delivery (health facilities) ought to be quite similar, allowing perhaps for differences of form but few if any differences of substance.

## Methods

The MEDLINE, EMBASE and HealthStar databases were searched for articles published in English using the keywords and search strategy described in Appendix 1, developed with the assistance of a medical librarian. To locate non–peer-reviewed reports, a keyword search was conducted in the following databases: (1) the International Health Faciliies Assessment Network (IHFAN) (2012), (2) The World Bank (2012) and (3) the World Health Organization (2012). Abstracts from all manuscripts retrieved in the MEDLINE/EMBASE search were screened and inclusion criteria applied. Non–peer-reviewed reports were initially obtained in full text when an abstract was unavailable and the same inclusion criteria applied. All manuscripts and reports meeting the inclusion criteria were included in the analysis.

The inclusion criteria were the presence of an assessment tool, checklist or questionnaire that evaluated the availability of health resources or services at the health facility level. All questionnaires, regardless of the level of care evaluated (health posts, health centres, hospitals, etc.), were included, consistent with WHO’s definition of health systems as being inclusive of ‘all organizations, people, and actions whose primary intent is to promote, restore or maintain health’ (World Health Organization 2007). We excluded commentaries, reports without a standardized assessment, assessments that evaluated only a specific type of health service (e.g. sexual and reproductive health, surgery, etc.) and any reports that lacked explicit methodologies for data collection, such as anecdotal accounts of health service availability. Authors whose studies made reference to systematic data collection tools or approaches but did not include them in the manuscript were contacted for copies of these assessments and clarification of the methods used.

**Figure 1:**
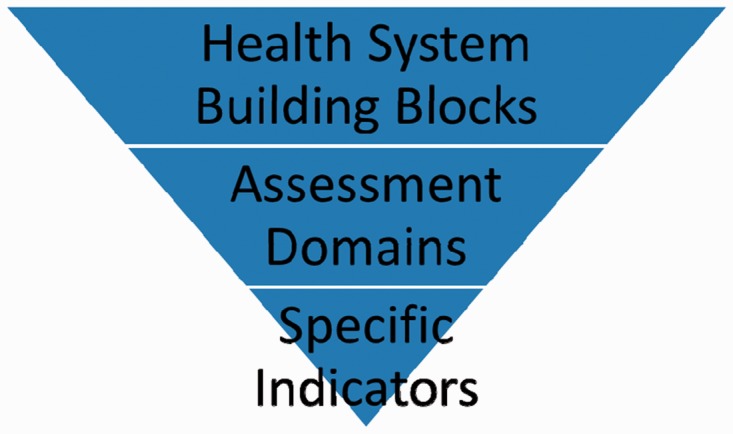
Hierarchy of terms

The analysis of these assessments was conducted using a health systems framework to contextualize them in the essential capacities of health systems in LMIC. We used the WHO’s health systems building blocks as an organizing framework (World Health Organization 2007) and treated the data hierarchically, beginning with the building blocks (Leadership/Governance; Health Care Financing; Health Workforce; Medical Products, Technologies; Information and Research; and Service Delivery). The WHO health systems framework and building blocks were selected because of the universality of the building blocks, the appropriateness of their use in LMICs, and the familiarity of the framework to practitioners in the field. Within each building block, thematic assessment domains were identified that corresponded to more specific indicators of health services or functions. For example, an indicator collecting the number of nurses present corresponded with the assessment domain of a health worker census within the health workforce building block. A graphical representation of this hierarchy is included in Figure 1.

Data were extracted into an Excel (Microsoft Corporation, USA) database from all included reports using a thematic analysis of the health services included in the assessment tools by one reviewer (JWN). All the tools were analysed twice: the first time to compile a list of the assessment indicators present in each of the tools and the second time to evaluate which of these indicators were measured by each of the assessment tools. Each extracted indicator was categorized into a broader domain reflecting a group of health services. For instance, an indicator measuring the availability of surgical instruments was included under the domain ‘surgery’. Each of these domains was then mapped to the health systems building blocks contained in the health systems framework. Using the defined list of assessment domains and their indicators, a second reviewer screened 30% of the assessment tools to ensure consistency in the data extracted. Disagreements in data extraction were resolved through discussions among two reviewers until consensus was achieved.

A recently published review of health facility assessment tools examined four of the assessments included in our study for the purpose of developing indicators of newborn care (Gabrysch *et al.* 2012). Although that review was focused on one type of health service (routine and emergency newborn care) and not on a general assessment of service availability, it provides an additional mechanism of comparing the results of our analysis with those of other authors. We compared our extracted results with the relevant indicators examined in that study and found that our data extraction matched theirs, with the exception of the safe administration of oxygen, which may be because of differences in the survey instrument used, providing an additional validation of our results.

## Results

### Study selection

**Figure 2:**
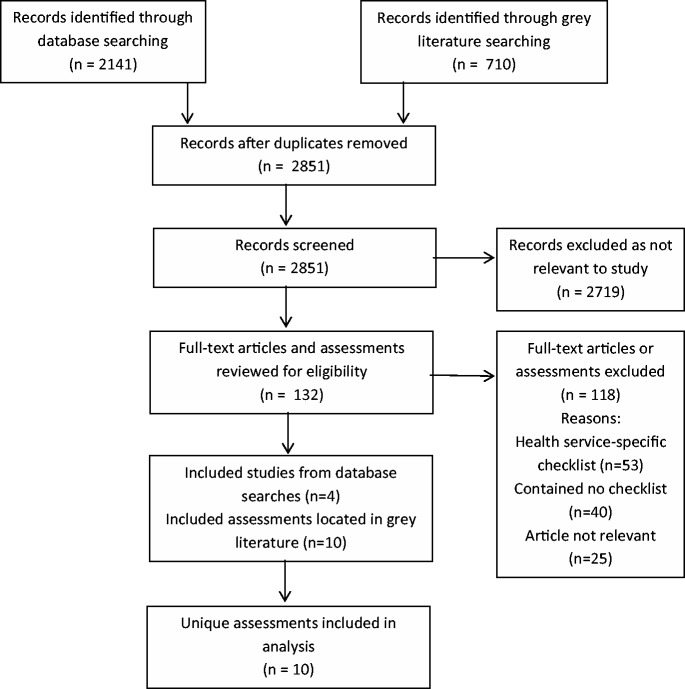
PRISMA flowchart

The search strategy was run on 5 May 2011 and updated for new results on 19 April 2012. A total of 1962 abstracts obtained in the first search of the MEDLINE, EMBASE and HealthStar databases were located and an additional 179 abstracts were retrieved following the second search. Of the abstracts screened, 121 full-text manuscripts were obtained and screened for eligibility, as well as 11 references from the other databases. Only four studies located in the medical literature databases reported using a standardized assessment tool thereby meeting the inclusion criteria. Of these, three reported using the Service Provision Assessment (Hong *et al.* 2006; Agha and Do 2009; Cherlin *et al.* 2011) and one reported using the Health Facility Census (Gabrysch *et al.* 2011); both of these tools were also located in the IHFAN database. None of the four studies included copies of the tools (Figure 2).

This search also located two other reviews of health facility assessment tools, one published by MEASURE Evaluation (Edward *et al.* 2009) and one by The World Bank (Lindelow and Wagstaff 2003), neither of which utilized a systematic literature search and both included fewer assessments than were located for this review. All the assessment tools included in these previous reviews had already been located through our search strategy.

### Characteristics of the included assessment tools

Ten health facility assessment tools were identified through the search strategy (Table 1). All of the assessment tools collected are intended for use in LMIC, with one (Health Resources Availability Mapping System—HeRAMS) intended for use during humanitarian emergencies (Global Health Cluster 2009). All of the included assessment tools were readily available online, with the exception of the Health Facility Census developed by the Japan International Cooperation Agency and the Afghanistan Balanced Scorecard (Edward *et al.* 2011), which were obtained by contacting the authors and requesting a copy of the assessment tools.

**Table 1:** Summary of included assessment tools

Assessment tool	Developed by	Version
1. Facility Audit for Core Indicators	MEASURE Evaluation/United States Agency for International Development (USAID)	2007
2. Service Provision Assessment (SPA)	MEASURE Evaluation/USAID	Uganda, 2007
3. Service Availability Mapping (SAM)	WHO	2009 (date when SAM was discontinued)
4. Balanced Score Card (BSC), F and H forms	Johns Hopkins University/Ministry of Public Health of the Islamic Republic of Afghanistan	July 2011
5. Health Facility Census	Japan International Cooperation Agency (JICA)	Zambia Health Facility Census, September 2007
6. Health Resources Availability Mapping System	WHO	Sudan, 2012
7. Service Availability Readiness Assessment (SARA)	WHO	October 2011
8. Living Standards Measurement Study	Vietnam General Statistical Office	Vietnam, 1997–1998
9. Nigeria Public Delivery of Primary Health Care Services	National Primary Health Care Development Agency and World Bank	2002
10. Indonesian Family Life Survey (IFLS)	RAND Corporation	IFLS4, 2007

All of the tools utilized a checklist-like approach and the Afghanistan Balanced Scorecard supplemented quantitative data with qualitative interviews. The assessments varied considerably in format and length, highlighting the challenges in comparing data derived from different settings using different tools that have varying degrees of complexity.

### Thematic analysis of assessment domains

Our thematic analysis of the tools revealed 41 different assessment domains or sub-domains. We mapped these to the health systems building blocks contained in the health systems framework (Table 2) and provide a discussion of the included building blocks below.

**Table 2:** Framework of health facility assessment domains and subdomains with corresponding WHO health systems building blocks

Health systems building blocks	Assessment domains and sub-domains identified
1. Leadership/Governance	Ownership/management of facility
2. Health Care Financing	Financing of facility
	User fees charged/cost of service
3. Health Workforce	National health professions/cadres of workers
4. Medical Products, Technologies	Basic equipment
	Diagnostic/imaging services
	Laboratory services
	Pharmacy
	Essential medicines
	Nutrition
5. Information and Research	Service utilization
	Disease registers
	Caseload data
	Mortality data
	Vital statistics
	Evidence-based guidelines
	Continuing medical education
6. Service Delivery	Basic structural components
	Identification as a health facility
	Bed census
	General clinical services
	Non-communicable diseases
	Child health
	Outpatient Department/emergency room
	Dental/oral health
	Communicable diseases
	HIV/AIDS
	Vaccines
	Infection control
	Cleaning/sterilization
	Sexual and reproductive health
	Obstetric care
	Sexually transmitted infections
	Surgery
	Intensive care unit
	Disabilities and injury rehabilitation
	Mental health care
	Internal medicine
	Palliative care
	Mortuary
	Environmental health
	Nutrition

None of the tools included in our review collected data on all the assessment domains we identified and the specificity of the tools varied significantly, ranging from indicators in 13 to 33 (median: 25.5) of the 41 assessment domains. The majority of the differences fell within the areas of health services delivery, with considerable variation in the types of health services assessed and with a preference towards assessments of services at the primary care or community level rather than secondary-level services such as surgery or intensive care. A summary of the assessment domains and tools is included in Table 3.

**Table 3:** Summary of included assessment tools

Health system building blocks and domains	MEASURE core indicators	Service provision assessment	Service availability mapping	Balanced score card	Health facility census	Health resources availability mapping system	Service availability readiness assessment	Living standards measurement study	Nigeria public delivery of primary health care services	Indonesian family life survey
Leadership/Governance										
Ownership/management of facility		✓	✓	✓	✓	✓	✓		✓	✓
Health Care Financing										
Financing of facility		✓				✓				
User fees charged		✓				✓		✓	✓	✓
Health Workforce										
Health human resources (cadres of health workers)	✓	✓	✓	✓	✓	✓	✓	✓	✓	✓
Medical Products, Technologies										
Basic equipment	✓	✓	✓	✓	✓		✓	✓	✓	✓
Diagnostics/imaging		✓	✓	✓	✓	✓	✓			✓
Laboratory services	✓	✓	✓	✓	✓	✓	✓	✓	✓	✓
Pharmacy		✓		✓	✓					✓
Essential medicines	✓	✓	✓	✓	✓		✓		✓	✓
Information and Research										
Collection of vital statistics	✓	✓		✓		✓				
Caseload data	✓	✓		✓	✓	✓	✓			✓
Priority disease caseload register	✓	✓	✓	✓	✓					
Evidence-based guidelines	✓	✓	✓	✓	✓		✓			✓
Continuing medical education		✓	✓	✓			✓			✓
Service Delivery										
Basic structural components	✓	✓	✓	✓	✓	✓	✓	✓	✓	✓
Bed census		✓	✓	✓	✓	✓	✓		✓	
Identification as a health facility					✓	✓				
General clinical services	✓	✓	✓	✓	✓	✓	✓		✓	✓
Non-communicable diseases					✓	✓	✓			✓
Child health	✓	✓	✓	✓	✓	✓	✓	✓	✓	✓
Outpatient department/Emergency room	✓	✓		✓	✓	✓				
Dental/oral health		✓			✓			✓	✓	✓
Communicable diseases	✓	✓	✓	✓	✓	✓	✓		✓	✓
HIV/AIDS	✓	✓	✓	✓	✓	✓	✓			
Vaccines		✓	✓	✓	✓	✓	✓		✓	✓
Infection control	✓	✓	✓	✓	✓	✓	✓			✓
Cleaning/sterilization	✓	✓	✓	✓	✓	✓	✓	✓	✓	✓
Sexual and reproductive health	✓	✓	✓	✓	✓	✓	✓	✓	✓	✓
Obstetric care	✓	✓	✓	✓	✓	✓	✓	✓	✓	✓
Sexually transmitted infections	✓	✓	✓		✓	✓	✓		✓	✓
Surgery	✓		✓	✓	✓	✓	✓	✓		✓
Intensive care unit				✓	✓					
Disabilities and injury rehabilitation					✓	✓				
Mental health care					✓	✓				
Internal medicine					✓					
Palliative care	✓	✓			✓		✓			
Mortuary					✓					
Environmental health					✓	✓	✓			
Nutrition		✓			✓	✓	✓	✓		

### Health systems building blocks

#### Leadership/governance

Eight of the included assessments contained some form of basic information concerning the organization, ownership and leadership of health facilities. These data consisted of basic descriptive data of the ownership of the facility, department heads and basic questions of how the facility was organized and run. While these data were frequently available, they were generally very basic indicators of ownership or leadership of the facility and provided little in the way of measuring the quality of leadership, which may well be beyond the scope of these tools.

#### Health care financing

Only two assessments collected information on how the health facility was financed and only five collected information on whether user fees were charged. Countries where the health system is financed through governmental schemes may account for the absence of these questions in some assessments (such as in Afghanistan; Sabri *et al.* 2007); however, assessments of donor or organizational funding for individual facilities provide a nuanced assessment of the sustainability of the health system.

#### Health workforce

All the assessments collected some data on health human resources, disaggregated by specific cadres of health professionals, although these cadres varied significantly among the assessments. Only five of the assessments contained indicators for assessing the hours of operation of the facility (when staff would be present) and only three collected data on the availability of emergency staff in-house 24 hours a day.

#### Medical products, technologies

Significant variation was uncovered in the assessment of diagnostic services, essential medicines and laboratory services. All of these domains have the potential for expansive lists of assessment criteria (consider every medication on a country’s essential medicines list or every diagnostic test available), although most used a selective sampling rather than an exhaustive list. For example, most assessments included indicators for basic equipments such as a stethoscope, blood pressure cuff and adult scale.

Assessments of diagnostic services (including laboratory and diagnostic imaging) were frequently included, although again the specificity of these assessments varied. All 10 of the assessment tools evaluated the availability of some form of laboratory services, while only 7 assessed the availability of diagnostic imaging. These assessments ranged from indicators for specific analytical tests and equipment to general questions of capacity (e.g. ‘Are malaria diagnostic services available at this health facility?’).

#### Information and research

Our results located two kinds of information and research of relevance to this building block: health information systems and clinical practice guidelines.

Eight of the included assessments in our review contained assessments of different aspects of health information systems. Caseload data (including for priority diseases identified, such as HIV or tuberculosis) were the most frequently collected and were included in eight of the tools, while assessments of communicable disease surveillance systems were the second most frequently collected data in six of the tools. Other data collected were less consistent across the assessments, such as vital statistics, patient charts and vaccination activity.

Seven of the included assessments contained indicators for assessing the availability of evidence-based guidelines for relevant health conditions. These guidelines were often integrated into assessments of service availability, assessing services for the availability of essential resources and equipment as well as relevant clinical practice guidelines. Five of the included tools assessed whether clinicians had received continuing medical education or training in specific areas, generally over the past two years.

#### Service delivery

The delivery of health services is the point of contact between patients and the health care system, where diagnosis and treatment occur. Not surprisingly, service delivery accounts for the bulk of the data collected by the assessments included in our review, collected across a large number of different clinical domains. The range of clinical services evaluated are in some ways misleading as some tools included only single indicators of complex, yet poorly defined packages of services rather than specific measures (e.g. ‘family planning’ as one indicator rather than a series of individual service or process indicators).

All of the assessment tools also included some form of basic structural assessments, such as the condition of walls, floors and heating/cooling systems. As a basic structure constitutes an integral component of a health facility, this is included as an element of the health services delivery building block. Another key infrastructure assessment, the hospital bed census, was included in six of the included assessment tools, representing a significant absence of basic structural information in these tools.

Nutrition services were included in five assessments, including indicators for malnutrition screening services and therapeutic feeding. Environmental health services were less frequently included in assessment tools, with only three of the included assessments containing relevant indicators. One assessment included indicators for the availability of water and food inspection services, although this was not included in any others, nor does it seem a particularly relevant focus for health facilities. The other two assessments that included environmental health services were both related to malaria bed net distribution.

## Discussion

Health system strengthening requires reliable, accurate and comparable data sources across the health system to identify gaps in coverage and to identify priority health needs. The monitoring and evaluation of the health system necessitate a focus on how inputs and processes (e.g. health human resources and health services delivery) contribute to outputs (coverage of health services) and their impact on relevant health indicators (morbidity and mortality). The absence of any of these data sources results in incomplete information and unreliable assessments of priority areas (Echoka *et al.* 2013). Ensuring that these data are comparable across countries requires methodological consistency, which this review reveals does not currently exist. Among the various health system assessment tools used today, there are many substantive disagreements, which it stands to reason would introduce systemic confounding when carrying out health system assessments.

Two major concerns arise from the results of this review. First, and most importantly, the data being collected at the health facilities level are inconsistent, incomplete and incomparable among different tools and therefore different regions where these assessments are employed. At the level of the health facility, this has major implications for ensuring that the population has access to a comprehensive package of health services: the neglect of certain essential health services from some assessments paints an incomplete picture of the ability of a health facility to provide essential health services, potentially resulting in major gaps in the coverage of vital health services that are currently undetected and thereby failing to stimulate meaningful interventions to scale up the capacity of hospitals or clinics. More distally, this affects resource allocation from donors and ministries of health, which may result in system-wide failures to adequately plan for health needs.

Internationally, the heterogeneity of the assessments makes comparisons of gains in health systems strengthening or health service coverage difficult, if not impossible, to estimate reliably. While there has been a global movement towards improving the quality and quantity of individual health data collected (specifically vital statistics like births and deaths), this momentum appears to have left much of the essential health systems data behind. The results of our literature review located only four publications that implemented a standardized assessment in peer-reviewed publications; this low number may be the result of our search strategy, but it appears that there is either little awareness or little use of the tools currently in existence. A likely explanation is that although there is extensive experience in using these assessments by donors and ministries of health, the evidence base for these tools appears to be severely lacking and appears to be based on a restricted set of preferences or priorities rather than on a rigorous evaluation of the essential functions of health facilities or health systems.

Second, we noted a preference towards the evaluation of primary care services, with secondary and tertiary care being absent from many assessments, despite a need for these services in LMIC (Fowler *et al.* 2008). Furthermore, little data are available in the literature concerning the absence of or need for secondary and tertiary care infrastructure or services, potentially owing to the absence of these services in infrastructure assessments such as those included in our review.

A plausible explanation for this is that health systems have been shaped by international declarations such as the ‘Declaration on strengthening district health systems based on primary health care’ (the ‘Harare Declaration’) or the Alma Ata Declaration (Meessen *et al.* 2014). Following these declarations, many health systems adapted to this renewed focus on primary care and the organization of health services around the concept of district hospitals and health clinics, which may influence the development of health facility assessment tools to correspond with these priorities. If this is true, then these tools are likely more reflective of assessments of vertical programmes than of comprehensive health systems interventions.

Some of the variation is likely attributable to the level of health care for which the assessment was designed (e.g. assessments focused on primary health care are unlikely to inquire about the presence of a trained surgeon), which may also reflect donor or organizational priorities oriented towards specific kinds of programmes. This is an important finding, as these tools appear designed to assess the programmes that donors fund rather than initiatives that are led and monitored by the countries themselves.

Only one of the tools included in our review (HeRAMS) made use of an explicit framework to guide the assessment of available health resources and services. While the HeRAMS framework is explicit, it also does not clearly link to a broader health systems framework. This is, in part, because of the nature of the tool’s initial deployment in the Darfur region of Sudan, an area of ongoing conflict rather than stable development.

### The health system building blocks and assessment domains

This review provides a systematic catalogue of the broad domains employed in the assessment of health facilities. This catalogue should provide a foundation for the development of more detailed assessment indicators based on these domains and with the goal of making future assessments reflective of the essential characteristics of well-functioning health systems.

A significant number of gaps were uncovered that would likely lead to an incomplete assessment of the administrative or health service delivery functions of health facilities, which should raise serious concerns. While some building blocks are perhaps easier to quantify (the presence of an operating theatre, for example) than others (the quality of the management), each is important for ensuring that health systems evolve comprehensively and not disproportionately. Furthermore, many of the included indicators were evaluated differently, leading to concerns not only of incomparability among the tools, but also a lack of agreement of how best to measure specific functions or services.

Assessments of the leadership of a health facility, for example, often allow one to guess or infer other features of the facilities. A public hospital run by an international non-governmental organization may have access to different supply chains or human resources than a public hospital run by the ministry of health, for example. Combined with relevant geographic and population demographic information, this information allows for important analyses of equity in the distribution of health services and resources and the identification of underserved populations when health facilities and population data are compared (Noor *et al.* 2004; 2009; Moïsi *et al.* 2010). Closely linked to this, assessments of financial sustainability at the level of service delivery, where staff salaries are paid, infrastructure is upgraded or maintained and essential medicines and equipment purchased, among other costs is essential for understanding the impact of programmes on the system (Shakarishvili *et al.* 2010). 

Concerning health care financing, a standard dichotomy of public and private health services is pervasive in much of the literature, although the mix can be far more nuanced, including the contracting of health services by governments to other entities (Loevinsohn and Harding 2005). Regardless, the availability of information such as user fees and the costs for services may provide an important analytic tool for understanding barriers to health services and the potential economic impact of out-of-pocket health expenses on population health that can be collected at the health facility level. That only half of the included assessment tools collected information on user fees (the most pragmatic health expenditure for patients) represents a significant weakness.

Assessments of health human resources must necessarily focus broadly on ‘all people engaged in actions whose primary intent is to enhance health’ including clinical staff such as physicians, nurses and midwives, as well as management and support staff who do not provide direct clinical services, but who are essential in supporting the delivery of health services and the functioning of the health system (World Health Organization 2006). Including reliable estimates of staffing levels is therefore essential for providing one estimate of the capacity of the health facility.

In many LMIC, levels of health professionals have reached crisis levels as a result of a complex set of circumstances, ranging from supply–demand imbalances to internal, regional and international migration and labour market factors (Narasimhan *et al.* 2004). It can therefore not be assumed that staffing levels are adequate for sustaining health programmes; monitoring the availability of a country’s health workforce is integral for understanding the human resources available to deliver essential health services and should be a central component of health facilities assessments. International classifications of health workers exist and ought to form the basis of assessment criteria so as to allow for international comparisons to be drawn (International Labour Organization 2012) and ideally assessments of the health workforce should be mapped to these international classifications (World Health Organization 2010).

The foundations of public health presuppose the ability to quantify and monitor relevant health indicators of the population, making a functioning health information system essential for a functioning health system (AbouZahr and Boerma 2005). The information needs of health workers and those with responsibilities for leadership in the health system are broad and vast, including data on health needs, service utilization, vital statistics and surveillance of relevant communicable diseases. Within this, there is also a need for reliable evidence to guide treatment decisions and health resource planning. Regrettably, there are several constraints for accessing reliable information in LMIC, including the absence of evidence to support locally relevant medical conditions, as much of the body of medical knowledge has focused on priority illnesses in high-income countries (Ehrhardt and Meyer 2011; Chinnock *et al.* 2005). Paradoxically, clinicians in LMIC where resources are more limited have a greater need for evidence to ensure that the care provided does not waste scarce resources on incorrect diagnoses and ineffective treatments (Volmink *et al.* 2001).

We identified significant weaknesses related to service delivery, as several integral health services remained absent from most tools and the indicators to measure common services are frequently different, meaning many data are likely incomparable among the various tools. For example, non-communicable diseases, physical rehabilitation, mental health and palliative care were only assessed in a small number of the included assessments. Given that many of these conditions are underfunded and poorly accessible in LMIC, in general, it is not entirely surprising that they were largely neglected. Yet, even obviously necessary services were neglected by many assessments: the availability of a mortuary, for example, was included in only one assessment. Basic structural resources indicators were frequently included and appear to be of value in providing estimates of health facility capacity. For example, bed availability data are critical during emergency situations, provide a measure of a facility’s capacity and size, and when data on bed usage are also collected, provide a means of monitoring service utilization (Kanter and Moran 2007).

Many health services have developed specific assessments that apply a more detailed approach using specific indicators that correspond to assessments of the quality of processes, as well as the availability of essential infrastructure. For example, WHO has developed the Tool for Situational Analysis to Assess Emergency and Essential Surgical Care, which contains an extensive set of indicators and which has been successfully used in several countries (Choo *et al.* 2010). Other approaches have used a select set of signal functions as a proxy for more in-depth assessments, such as for Basic and Comprehensive Emergency Obstetric Care (WHO, UNFPA, UNICEF, AMDD 2009), routine and emergency newborn care (Gabrysch *et al.*, 2012) and for identifying essential components of health information systems (AbouZahr and Boerma 2005). A tracer medicines approach has been used for evaluating access to essential medicines, including 14 tracer medicines in use worldwide and 16 regionally specific medicines. Countries are further encouraged to collect data on 20 additional medicines of national importance, for a total of approximately 50 medicines to provide data on the drug supply system (World Health Organization 2010). Such an approach allows for an estimation of capacity, without extensive and laborious measurement. This standardized, universal approach to health service-specific evaluations may present an option for integrating more nuanced information into broader health facilities datasets and should be explored further in the pursuit of developing a standard assessment tool and set of indicators.

Considering this long list of potential health services, it is likely that our cataloguing of health services may be incomplete or is the subject of debate among other experts. Vision services, for example, were lacking among the assessments, with the exception of questions related to the availability of a vision chart and an ophthalmoscope in the Balanced Score Card. This was not considered to be an assessment of the provision of vision or ophthalmologic services, but rather the availability of basic equipment or the provision of general clinical services. It appears likely that other services have also been neglected and their inclusion in the evolution of this cataloguing of essential health services would be welcome.

### Study limitations and areas for future research and development

This study evaluated only those tools applied in LMIC and excluded search strategies that would have captured similar tools developed for use in high-income countries. The basis of this decision was that the health priorities of high-income countries are different from LMIC and that these priorities would be reflected differently in any assessment tool for high-income countries. For example, communicable diseases play a prominent role in many of the included assessments; while these diseases are of obvious concern in all countries, the particular emphasis on HIV, malaria and tuberculosis that is of heightened relevance in LMIC would not likely be as prominent in an assessment of health services availability in most high-income countries.

The decision to limit the search strategy to only those assessments that included a comprehensive assessment of all health services may have excluded some service-specific assessments that collected data pertinent to other health sectors. For example, an assessment that specifically targeted HIV treatment facilities may have collected data on general clinical services, sexual and reproductive health care and palliative care, among others. However, the goal of this study was not to catalogue the number of tools that collected data on given assessment domains (although that was a resultant outcome), but rather to generate a framework of essential assessment domains, linked to health systems building blocks, that could be applied in the development of future assessments. To that end, we feel justified in excluding these service-specific assessments, in favour of this more comprehensive approach.

Further studies that expand on this work could also consider including additional databases for their search, which may result in additional references being located in peer-reviewed journals and in the grey literature. This project’s findings are representative of the search strategy employed and expanding on the databases or keywords may result in additional sources, such as those in languages other than English.

Our approach utilized a broad health systems framework as the foundation for aligning health facilities assessment domains with a broader objective of health systems strengthening. In doing so, we have identified common domains that ought to be included as part of a health facilities census, which should guide the development of more specific assessment indicators to correspond to each of these domains similar to those service-specific tools mentioned above. Our review and recommendations fall short of prescribing particular indicators or assessment strategies; rather, we propose this as the next step in the evolution of these assessments: defining the indicators that best align with countries’ packages of health services and the information needs of specialists working in these areas of health service delivery to ensure that detailed assessments of specialized health services are comparable among countries.

Our results should be interpreted to recognize that different agencies (including national ministries of health) have a desire to exert ownership of their own data collection tools and processes, structured in a way that makes sense for the delivery or support of their own programmes. Rather than proposing the development of one tool that should be universally applied, our results propose a broad framework to be populated with internationally accepted indicators and basic datasets that can be used to guide the development of these tools, thereby ensuring a more comprehensive and coherent approach.

## Conclusions

This review highlights a fundamental problem in the collection of health facilities and health services availability data: the absence of common assessment tools yields incomparable data making it difficult, if not impossible, to track progress towards increasing access to health services, globally. Our review found 10 different health facility assessment tools currently in use. Our comparative analysis of these tools revealed that there are significant gaps in the areas evaluated by many of them, often orienting their focus towards primary care rather than the broader health system.

This review provides a framework in the form of 41 assessment domains linked to health systems building blocks that should guide the development of new health facilities assessment tools and the refinement of existing ones. Furthermore, these assessment domains provide a useful starting point for defining more detailed assessments that correspond to specialized health services. Future developments in this area should integrate existing specialized indicators into assessment tools to enhance the comparability of the data collected and to align these data with existing standards.

## Funding 

No specific funding was obtained for this project. Jason Nickerson was supported by a University of Ottawa Admission Scholarship and an Ontario Ministry of Training, Colleges, and Universities Ontario Graduate Scholarship. Amir Attaran was supported by a Social Sciences and Humanities Research Council Canada Research Chair. Peter Tugwell was supported by a Canadian Institutes for Health Research Canada Research Chair.

*Conflict of interest statement*. None declared.

## Appendix 1*:* MEDLINE/EMBASE literature search strategy

1. exp ‘Delivery of Health Care’/
2. health resources/ or ‘health services needs and demand’/ or health services accessibility/ or healthcare disparities/
3. exp Health Planning/
4. 1 or 3
5. exp ‘Equipment and Supplies’/
6. exp Health Facilities/
7. exp Health Services/
8. exp Health Manpower/
9. exp Health Personnel/
10. 6 or 7 or 8 or 9
11. Supply & Distribution.fs.
12. 10 and 11
13. 5 or 12
14. 4 and 13
15. service$ provision assessment.ab,ti.
16. service$ availability mapping.ab,ti.
17. (health facilit$ adj (census or assessment$ or map$)).ab,ti.
18. health resource$ avail$.ab,ti.
19. 15 or 16 or 17 or 18
20. 14 or 19
21. Developing Countries/
22. exp africa/ or exp caribbean region/ or exp central america/ or exp latin america/ or exp south america/ or exp asia, central/ or exp asia, southeastern/ or exp asia, western/ or exp china/ or ‘democratic people's republic of korea’/ or mongolia/ or exp indian ocean islands/ or exp melanesia/ or exp micronesia/ or exp polynesia/
23. 21 or 22
24. 20 and 23
25. 4 and 12
26. 19 or 25
27. 23 and 26
28. remove duplicates from 27

## References

[czu043-B1] AbouZahr C, Boerma T (2005). Health information systems: the foundations of public health. Bulletin of the World Health Organization.

[czu043-B2] Adam T, de Savigny D (2012). Systems thinking for strengthening health systems in LMICs: need for a paradigm shift. Health Policy and Planning.

[czu043-B3] Adam T, Hsu J, de Savigny D (2012). Evaluating health systems strengthening interventions in low-income and middle-income countries: are we asking the right questions?. Health Policy and Planning.

[czu043-B4] Agha S, Do M (2009). The quality of family planning services and client satisfaction in the public and private sectors in Kenya. International Journal for Quality in Health Care.

[czu043-B5] Attaran A (2005). An immeasurable crisis? A criticism of the millennium development goals and why they cannot be measured. PLoS Medicine.

[czu043-B6] Atun R (2012). Health systems, systems thinking and innovation. Health Policy and Planning.

[czu043-B8] Bosch-Capblanch X, Lavis JN, Lewin S (2012). Guidance for evidence-informed policies about health systems: rationale for andchallenges of guidance development. PLOS Medicine.

[czu043-B9] Casey S, Mitchell K, Mulamba Amisi I (2009). Use of facility assessment data to improve reproductive health service delivery inthe Democratic Republic of the Congo. Conflict and Health.

[czu043-B10] Cherlin EJ, Allam AA, Linnander EL (2011). Inputs to quality: supervision, management, and community involvement in health facilities in Egypt in 2004. BMC Health Services Research.

[czu043-B11] Chinnock P, Siegfried N, Clarke M (2005). Is evidence-based medicine relevant to the developing world?. PLoS Medicine.

[czu043-B12] Choo S, Perry H, Hesse AAJ (2010). Assessment of capacity for surgery, obstetrics and anaesthesia in 17 Ghanaian hospitals using a WHO assessment tool. Tropical Medicine and International Health.

[czu043-B13] Echoka E, Kombe Y, Dubourg D (2013). Existence and functionality of emergency obstetric care services at district level in Kenya: theoretical coverage versus reality. BMC Health Services Research.

[czu043-B14] Edward A, Matsubiyashi T, Fapohunda B, Becker S (2009). A comparative analysis of select health facility survey methods applied in low and middle income countries [working paper WP-09-11].

[czu043-B15] Edward A, Kumar B, Kakar F (2011). Configuring balanced scorecards for measuring health system performance: evidence from 5 years’ evaluation in Afghanistan. PLoS Medicine.

[czu043-B16] Ehrhardt S, Meyer CG (2011). Transfer of evidence-based medical guidelines to low- and middle-income countries. Tropical Medicine and International Health.

[czu043-B17] Falade CO, Oladoyinbo SO, Elemile TT (2006). How well equipped are healthcare facilities to manage childhood malaria? The situation in selected local government areas in South Western Nigeria. African Journal of Medicine and Medical Sciences.

[czu043-B18] Fowler R, Adhikari N, Bhagwanjee S (2008). Clinical review: critical care in the global context—disparities in burden of illness, access, and economics. Critical Care.

[czu043-B19] Frenk J (2010). The global health system: strengthening national health systems as the next step for global progress. PLoS Medicine.

[czu043-B20] Gabrysch S, Civitelli G, Edmond KM (2012). New signal functions to measure the ability of health facilities to provide routine and emergency newborn care. PLoS Medicine.

[czu043-B21] Gabrysch S, Cousens S, Cox J, Campbell OMR (2011). The influence of distance and level of care on delivery place in rural Zambia: a study of linked national data in a geographic information system. PLoS Medicine.

[czu043-B22] Global Health Cluster (2009). Health Resources Availability Mapping System Checklist.

[czu043-B24] Hong R, Montana L, Mishra V (2006). Family planning services quality as a determinant of use of IUD in Egypt. BMC Health Services Research.

[czu043-B25] http://www.ihfan.org/home/index.php?editable=yes&page_type=catalogsurveys#.

[czu043-B26] http://www.who.int/classifications/ME_component_nationalhealthplans_prepub_july2011.pdf.

[czu043-B27] http://www.ilo.org/public/english/bureau/stat/isco/isco08/index.htm.

[czu043-B28] Kanter RK, Moran JR (2007). Hospital emergency surge capacity: an empiric New York statewide study. Annals of Emergency Medicine.

[czu043-B30] Lindelow M, Wagstaff A (2003).

[czu043-B31] Loevinsohn B, Harding A (2005). Buying results? Contracting for health service delivery in developing countries. The Lancet.

[czu043-B32] Meessen B, Malanda B, for the Community of Practice “Health Service Delivery” (2014). No universal health coverage without strong local health systems. Bulletin of the World Health Organization.

[czu043-B33] Moïsi JC, Gatakaa H, Noor AM (2010). Geographic access to care is not a determinant of child mortality in a rural Kenyan setting with high health facility density. BMC Public Health.

[czu043-B34] Murray CJL, Frenk J (2000). A framework for assessing the performance of health systems. Bulletin of the World Health Organization.

[czu043-B35] Narasimhan V, Brown H, Pablos-Mendez A (2004). Responding to the global human resources crisis. The Lancet.

[czu043-B36] Noor A, Alegana V, Gething P, Snow R (2009). A spatial national health facility database for public health sector planning in Kenya in 2008. International Journal of Health Geographics.

[czu043-B37] Noor AM, Gikandi PW, Hay SI, Muga RO, Snow RW (2004). Creating spatially defined databases for equitable health service planning in low-income countries: the example of Kenya. Acta Tropica.

[czu043-B38] Paxton A, Bailey P, Lobis S, Fry D (2006). Global patterns in availability of emergency obstetric care. International Journal of Gynaecology and Obstetrics.

[czu043-B39] Sabri B, Siddiqi S, Ahmed AM, Kakar FK, Perrot J (2007). Towards sustainable delivery of health services in Afghanistan: options for the future. Bulletin of the World Health Organization.

[czu043-B40] Samb B, Desai N, Nishtar S (2010). Prevention and management of chronic disease: a litmus test for health-systems strengthening in low-income and middle-income countries. The Lancet.

[czu043-B41] Shakarishvili G, Lansang MA, Mitta V (2010). Health systems strengthening: a common classification and framework for investment analysis. Health Policy and Planning.

[czu043-B42] Shengelia B, Tandon A, Adams OB, Murray CJL (2005). Access, utilization, quality, and effective coverage: an integrated conceptual framework and measurement strategy. Social Science and Medicine.

[czu043-B44] Swanson RC, Cattaneo A, Bradley E (2012). Rethinking health systems strengthening: key systems thinking tools and strategies for transformational change. Health Policy and Planning.

[czu043-B45] Task Force on Health Systems Research (2004). Informed choices for attaining the millennium development goals: towards an international cooperative agenda for health-systems research. The Lancet.

[czu043-B46] The World Bank (2012). Central Microdata Catalog.

[czu043-B47] Travis P, Bennett S, Haines A (2004). Overcoming health-systems constraints to achieve the millennium development goals. The Lancet.

[czu043-B49] Volmink J, Swingler G, Siegfried N (2001). Where to practise evidence-based medicine?. The Lancet.

[czu043-B50] World Health Organization, United Nations Population Fund, United Nations Children’s Fund, and Averting Maternal Death and Disability (2009). Monitoring Emergency Obstetric Care: A Handbook.

[czu043-B51] World Health Organization (2006). Working Together for Health. World Health Report 2006.

[czu043-B52] World Health Organization (2007). Everybody’s Business: Strengthening Health Systems to Improve Health Outcomes Available.

[czu043-B53] World Health Organization (2010). Monitoring the Building Blocks of Health Systems: A Handbook of Indicators and Their Measurement Strategies.

[czu043-B54] World Health Organization World Health Organization.

